# Psychometric properties of the Malay version of the Women’s Views of Birth Labour Satisfaction Questionnaire using the Rasch measurement model: a cross sectional study

**DOI:** 10.1186/s12884-020-02975-z

**Published:** 2020-05-14

**Authors:** Mohd Noor Norhayati, Adnan Fatin Imtithal, Mat Junoh Nor Akma

**Affiliations:** Department of Family Medicine, School of Medical Sciences, Health Campus, Universiti Sains Malaysia, 16150 Kubang Kerian, Kelantan Malaysia

**Keywords:** Labor, Postpartum, Psychometric properties, Rasch model, Satisfaction

## Abstract

**Background:**

Birth satisfaction represents a complex construct of implicit and profound relevance to a woman’s perceived birth experience. It correlates with the childbearing woman’s experience of the quality of care received and stress during labor. This study aimed to examine the psychometric properties of the Malay language version of the Women’s Views of Birth Labour Satisfaction Questionnaire (WOMBLSQ) on labor satisfaction using the Rasch rating scale model.

**Methods:**

A cross-sectional study was conducted. The translated Malay version of the WOMBLSQ was completed by 200 postpartum women in a tertiary hospital. The Rasch model was applied to investigate the statistics, unidimensionality, item polarity and misfit, person misfit and person item distribution map.

**Results:**

The Rasch analysis showed that the 27 items, in nine dimensions, had high item reliability and item separation at 0.98 and 7.65 respectively, while good person reliability and person separation were at 0.78 and 1.90, respectively. Item 6 (*‘My birth partner/husband couldn’t have supported me any better’)* (outfit MnSq = 1.74, outfit z-std = 6.9, PtMea Corr = − 0.02) and Item 5 *(‘My birth partner/husband helped me to understand what was going on when I was in labor’)* (outfit MnSq = 1.65, outfit z-std = 2.9, PtMea Corr = 0.13) are misfit. Item 6 needs to be re-examined for removal or rephrasing, while Item 5 correlates poorly with the construct. Eight persons have the most misfitting response strings based on Item 6 but extremely trivial differences were found in the parameter estimates after refitting the model. Ten items easily endorse satisfaction from the respondents.

**Conclusion:**

The WOMBLSQ tested among postpartum women has been shown to have a good person reliability index and a high item reliability index. Items 5 and 6 do not contribute in the construction of scale but not degrading and suggested for refining. The spread of item difficulty should be improved in the future modification of items.

## Background

Maternal satisfaction refers to a woman’s feelings about her healthcare providers, the quality of communication and care received during childbirth, and health-related outcomes [1]. Continuous support and close relationships with maternity providers contribute to maternal birth satisfaction. Birth satisfaction is profoundly relevant to a woman’s perception of her experience of giving birth [2]. This perception is associated with the quality of care during childbirth and stress [3].

Various instruments have been developed to assess women’s satisfaction with labor and childbirth. These have all been designed and standardized for groups of English-speaking women, such as the Satisfaction with Antenatal Care Questionnaire, Satisfaction with Intrapartum Care and Satisfaction with Postnatal Care Questionnaire [4], the Maternal Satisfaction Questionnaire [5], and the Women’s Views of Birth Labour Satisfaction Questionnaire (WOMBLSQ) [6]. These questionnaires assess the woman’s satisfaction with childbirth as a multidimensional construct, with each dimension including various aspects relevant to measuring satisfaction [7]. Among the available scales, WOMBLSQ is multidimensional and sensitive to differences in settings and between women. It has the capacity to reflect the most relevant aspect of maternal satisfaction with care during labor. It can be utilized to compare and contrast satisfaction with different models of care or configurations of services or to assess changes over time [8].

Understanding maternal satisfaction is important for several reasons. First, maternal satisfaction is a proxy measure for quality of care. It reflects the quality of care during delivery in tertiary centers as well as the quality of care during the postpartum period, as provided by the primary healthcare facilities. Second, labor satisfaction influences the outcomes of the patient. A high level of satisfaction with the quality of care given by the caregivers during labor is associated with increased adherence, better continuity of care, and encouragement of the patient in the planning of maternity services and positive adjustment [9]. Third, in Malaysia, most studies have focused on satisfaction during antenatal care [10, 11], while others have focused on satisfaction with receiving spinal anesthesia during labor [12]. To our knowledge, there is a lack of questionnaires assessing labor satisfaction and this will be the first Malay language labor satisfaction questionnaire to be tested on the local population in Malaysia. We did not find any Malay versions of the questionnaire in our literature search, which was confirmed with the original author when asking for permission to use this questionnaire. Fourth, the assessment of patient satisfaction during labor, using a validated questionnaire in the Malay language, provides space for improved preventive strategies in the quality of obstetric services.

The aim of this study was to establish the psychometric properties of the Malay version of WOMBLSQ on labor satisfaction using the Rasch rating scale model. We used the Rasch analysis to observe the pattern of item responses among postpartum women because 98% of the population in this setting are Malays who speak the Malay language. We hypothesized that the Malay version of WOMBLSQ is a valid and reliable tool to assess maternal satisfaction during childbirth. The Rasch analysis, which is based on item response theory, is probabilistic and inferential, and focuses on the pattern of item responses that stipulate the interaction between a person and an item based on a mutual latent trait [13]. It predicts the likelihood of how a person with a different ability level for a particular trait would respond to an item of a certain level of difficulty. The probability of success depends on the difference between the ability of the person and the difficulty of the item [14].

## Methods

### Population and sample

A cross-sectional study took place between July and August 2017. The study was performed among postpartum women at the time of their discharge from a tertiary hospital in Kota Bharu, Kelantan. Women aged 18 years and above, with singleton pregnancies, who had undergone vaginal deliveries and were able to speak and understand the Malay language were included. Non-Malaysian citizens, those admitted only for medical observation and those with medical illnesses, were excluded. Convenient sampling was applied. The sample size was based on ½ logit at 99% confidence with best to poor targeting sample size between 108 and 243 [15] and the sample recruited for this study was 200 women following childbirth.

### Research tools

#### Women’s views of birth labour satisfaction questionnaire

This questionnaire measures maternal satisfaction with care received following childbirth. The English version of WOMBLSQ includes 11 dimensions with 32 items, which are rated on a 7-point Likert scale from “totally disagree” to “totally agree”. The dimensions were professional support (5 items), expectations (4 items), home assessment (3 items), holding baby (3 items), support from husband / partner (3 items), pain in labor (3 items), pain after delivery (3 items), continuity (2 items), environment (2 items), control (2 items), and general satisfaction (2 items). The items were deliberately very positive or negatively worded (Q4, Q6, Q8, Q9, Q10, Q15, Q16, Q18, Q21, Q25, Q26, Q28, Q29, Q30) to enhance the respondent’s ability to express minimal dissatisfaction. Dimension scores were generated to allow easy comparisons between individual dimensions and the total score was generated by adding the scores of all the items. The scores were added with the negatively worded items being reversed and transformed to percentage score so that the minimum possible score is 0 and the maximum possible score is 100%. Higher scores indicate greater satisfaction. It demonstrated adequate reliability on a global scale with a Cronbach’s alpha of 0.89, and for the subscales the Cronbach’s alpha values ranged between 0.62 and 0.91 [6].

#### Translation process

In order to ensure a high quality of translation, it was conducted in accordance with the recommended ten steps of Principles of Good Practice for the Translation and Cultural Adaptation Process. It began before the translation with preparation that included contacting the developer for permission to use the instrument. Forward translations from English to the Malay language were performed by a linguist and a medical doctor. The two forward translations were reconciled, and the first consensus was produced. Back translation of the first consensus was conducted into English by another linguist and medical doctor. A review was done of the back translation to compare the two translated versions of the questionnaire to produce an interim version. Harmonization is referring to comparing the translated and the original versions. Cognitive debriefing was performed by testing the questionnaire on six postpartum women to check for understanding and in order to test the understanding and its relevance in the tested culture. A comparison between the original and the layman versions were performed in order to highlight and amend discrepancies. Proofreading of the final review of the translation was done to highlight and correct any typographic, grammatical or other errors. The final report was produced at the end of the process documenting the development of each translation [16]. For the translated Malay version, we removed the home assessment dimension because it is not related to our local setting, and the general satisfaction domain was analyzed separately from other satisfaction domains [6]. Therefore, nine dimensions with 27 items were analyzed (for further information, please see the supplementary file).

### Data collection

During hospitalization, the postpartum women in obstetric wards were identified based on the study’s eligibility criteria. They were briefed and invited to participate in the study. Once the women voluntarily agreed to participate, informed consent was taken. They were recruited until the required sample size was met. The translated Malay version of WOMBLSQ on labor satisfaction was administered before their discharge from hospital, to ensure that they were fit and medically stable. Sociodemographic characteristics of the patients were also obtained. There was no foreseeable or potential risk that arose from participation in this research.

### Statistical analyses

Rasch rating-scale model was used to measure respondents’ EBM knowledge using a five-point Likert scale. The Rasch rating-scale model estimates the probability that a person will choose a particular response category or an item [14]. The Rasch analysis places persons and items on the same measurement scale where the unit of measurement is the logit.

Person and item reliability values of more than 0.8 are acceptable values, while values between 0.6–0.8 are less acceptable and values less than 0.6 are not acceptable [14]. The acceptable value of separation indices is more than 2.0, which leads to a value of > 0.8 for the corresponding person and item reliabilities [14, 17, 18]. Value of unexplained variance by 1st contrast (size) < 3.0 units is good, while < 5% is well accepted [19]. It is also suggested that the Eigenvalue of unexplained variance by 1st contrast < 3% is excellent, 3–5% is very good, 5–10% is good, 10–15% is fair and > 15% is poor [20]. Rasch analysis requires at least a minimum of 40% raw variance explained by measures [20] and is it better to exceed 60% [19]. Item dependency or multicollinearity is identified from the largest standardized residual correlation. Locally dependent items pairs which are highly correlated (> 0.7) can be considered as redundant.

An item is a misfit when it violates the goodness of fit criteria for point-measure correlation (PtMea Corr), outfit mean square (MnSq) and outfit z-score standardized (z-std) values. The working parameter for an acceptable PtMea Corr value shall be between 0.4 < PtMea Corr < 0.8 [14]. For politomous data (Likert scale) the acceptable range of fit items for Likert scale is between 0.5 logits to 1.5 logits, which indicate productive for construction of measurement scale. Values less than 0.5 indicate less productive but not degrading. Values between 1.5 and 2.0 indicates unproductive but not degrading and values more 2.0 will distorts or degrades the scale [21]. The outfit z-std values between − 2 and + 2 is acceptable [21]. Data analyses were conducted using Winsteps version 3.72.3 [22] for Rasch measurement.

## Results

A total of 200 postpartum women participated in this study by completing the questionnaire. All participants were married. The majority of the participants were aged below 40 years old. Other sociodemographic characteristics of the participants are presented in Table 1.

**Table 1:** Sociodemographic characteristics of participants (*n* = 200)

Variables	Mean (SD^a^)	n	(%)
Age (year)	28.9 (5.21)		
Number of children	2.0 (2.00)^b^		
Religion			
Muslim		195	(97.5)
Buddhist		5	(2.5)
Occupation			
Private		31	(15.5)
Government		51	(25.5)
Self-employment		32	(16.0)
Housewife		81	(40.5)
Others		5	(2.5)
Education			
Primary school		4	(2.0)
Secondary school		100	(50.0)
University		96	(48.0)

^a^ Standard deviation

^b^ Median (interquartile range)

### Person and item reliability

Table 2 shows the summary statistics of 200 measured persons and 27 measured items of Women’s Views of Birth Labour Satisfaction Questionnaire to examine the fit of the data to the Rasch model. A total of 5400 data points are analysed using Winsteps version: 3.72.3, Rasch Measurement software. This generated a log-likelihood chi-square value of 14,832.41 with 5169 degrees of freedom and *p* = 0.000. The Global Root-Mean Square Residual was 1.4163. The Cronbach Alpha (KR-20) Person Raw Score was at 0.78.

**Table 2:** Person and item summary statistics (initial analysis)

	Person (*n* = 200)	Item (*n* = 27)
Cronbach’s alpha (α)	0.78	
Reliability index (μ)	0.78	0.98
Separation index	1.90	7.65
Mean	0.54	0.00
Max measure	3.03	0.74
Min measure	−0.30	−0.91
Spread	3.33	1.65
Standard deviation	0.35	0.51
Outfit		
Mean Square	1.05	1.05
z-Standard	−0.10	0.10

The Item reliability, μ_item_ is 0.98 with standard error (SE) of 0.10. The indices indicate that the items are very good as the values are close to 1.0. The Person reliability, β is 0.78, which slightly matches the expected model of 0.83 with SE of 0.03.

### Separation index

The Item separation index is excellent at 7.65 (Table 2), indicating the person’s ability to discriminate the 27 items into seven strata or levels of agreement. While, the person separation index is 1.90.

### Unidimensionality

Unidimensionality is a crucial element in determining construct validity. In Rasch, it can be identified using the Principal Component Analysis (PCA) as depicted in Table 3. To satisfy unidimensionality, items in the instruments must measure the same composite of abilities i.e. knowledge on EBM. The Rasch PCA of residuals yields the raw variance explained by measures of 44.6%, which was close to variance expected by the model (43.9%). The unexplained variance in 1st contrast is 10.0%.

**Table 3:** Standardized residual variance using Principal Component Analysis

Standardised residual variance (in Eigenvalue units)	Empirical (%)
Total raw variance in observations	100.0
Raw variance explained by measures	44.6
Raw variance explained by persons	9.6
Raw variance explained by items	35.0
Raw unexplained variance (total)	55.4
Unexplained variance in 1st contrast	10.0

### Local item dependency

None of the items breach the 0.70 limit indicating item independence in the instrument (Table 4). For example, for most of respondents, for item 26 ‘*Everyone seemed to tell me what to do in labour’* has material effect on item 27 on *‘Q30: Labour was just a matter of doing what I was told by my carers’.* Both items were from the domain control.

**Table 4:** Largest standardized residual correlations for items

Correlation	Item	Item
0.59	Item 26	Item 27
0.52	Item 1	Item 2
0.50	Item 10	Item 27
0.47	Item 2	Item 3
0.45	Item 7	Item 27
0.40	Item 6	Item 13
0.40	Item 7	Item 18
−0.43	Item 6	Item 18
−0.41	Item 6	Item 27
−0.40	Item 9	Item 27

The response pattern is 59% similar between item 26 and item 27 (Table 5). At the highest level, the person giving response 7 for item 26 has given response 7 for items 27. Similarly, at the lowest level, the person giving response 4 for item 26, has given response 6 for item 27.

**Table 5:** Mapping of the largest residual correlations (0.59) of responses for item 26 and 27

Person					Item	
					26	27
4	1	4	2	3	7	7
1	1	3	4	2	6	7
1	1	1	4	2	7	7
5	1	7	4	2	7	7
2	1	1	1	3	7	7
--- consolidated ---						
1	1	1	4	2	4	4
4	1	1	2	3	3	3
3	1	1	1	3	3	2
2	1	2	3	2	2	3
2	1	1	3	3	4	6

### Goodness of fit test

The Item fit statistics investigation on overall outfit MnSq and outfit z-std show that the outfit MnSq was 1.05 and outfit z-std was 0.10 (Table 2). A further investigation into the item misfit statistics was conducted. The parameters for the 27 item statistics are as follows:

- Measure = 0.74 *logit* > x < − 0.89 *logit*
- Outfit MnSq = 1.74 *logit* > x < 0.63 *logit*
- Outfit z-std = 6.9 *logit* > x < − 4.80 *logit*
- PtMea Corr = 0.68 *logit* > x < − 0.02 *logit*

Based on item misfit order, item 6 (*‘My birth partner/husband couldn’t have supported me any better’)* (outfit MnSq = 1.74, outfit z-std = 6.9, PtMea Corr = − 0.02) and item 5 *(My birth partner/husband helped me to understand what was going on when I was in labour)* (outfit MnSq = 1.65, outfit z-std = 2.9, PtMea Corr = 0.13) are misfit.

### Person misfit

The Person fit statistics investigation on overall outfit MnSq and z-std showed that the outfit MnSq was 1.05 and outfit z-std was − 0.10, that is very near to expectation of 1.00 and 0.00 (Table 2). A further investigation into the person misfit statistics was conducted to ensure that the 200 persons were in fit conditions. Person fit were examined and the ranges of parameters for person are as follows:

- Measure = 1.54 *logit* > x < − 0.01 *logit*
- Outfit MnSq = 4.14 *logit* > x < 0.21 *logit*
- Outfit z-std = 4.40 *logit* > x < − 3.30 *logit*
- PtMea Corr = 0.86 *logit* > x < 0.00 *logit*

Upon further investigation, 8 persons were found to show most misfitting response strings based on item 6. These 8 persons were deleted because they were of no psychometric interest and add noise to the measurement process [17]. To evaluate the impact of misfitting, the EBM questionnaire was fitted twice; first, with all the items and persons; and second, eliminating 8 persons from the model. Based on our findings, extremely trivial differences were found in the parameter estimates (Table 6).

**Table 6:** Person and item summary statistics before and after removal of misfit responses

	Before identifying misfit respondents		After identifying misfit respondents	
	Person (n = 200)	Item (n = 27)	Person (*n* = 192)	Item (*n* = 27)
Cronbach’s alpha (α)	0.78		0.78	
Reliability index (μ)	0.78	0.98	0.79	0.98
Separation index	1.90	7.65	1.94	7.62
Mean	0.54	0.00	0.54	0.00
Max measure	3.03	0.74	3.10	0.74
Min measure	−0.30	− 0.91	−0.31	− 0.98
Spread	3.33	1.65	3.41	1.72
Standard deviation	0.35	0.51	0.36	0.52
Outfit				
Mean Square	1.05	1.05	1.04	1.04
z-Standard	−0.10	0.10	−0.10	0.00

### Wright distribution map

**Fig. 1:**
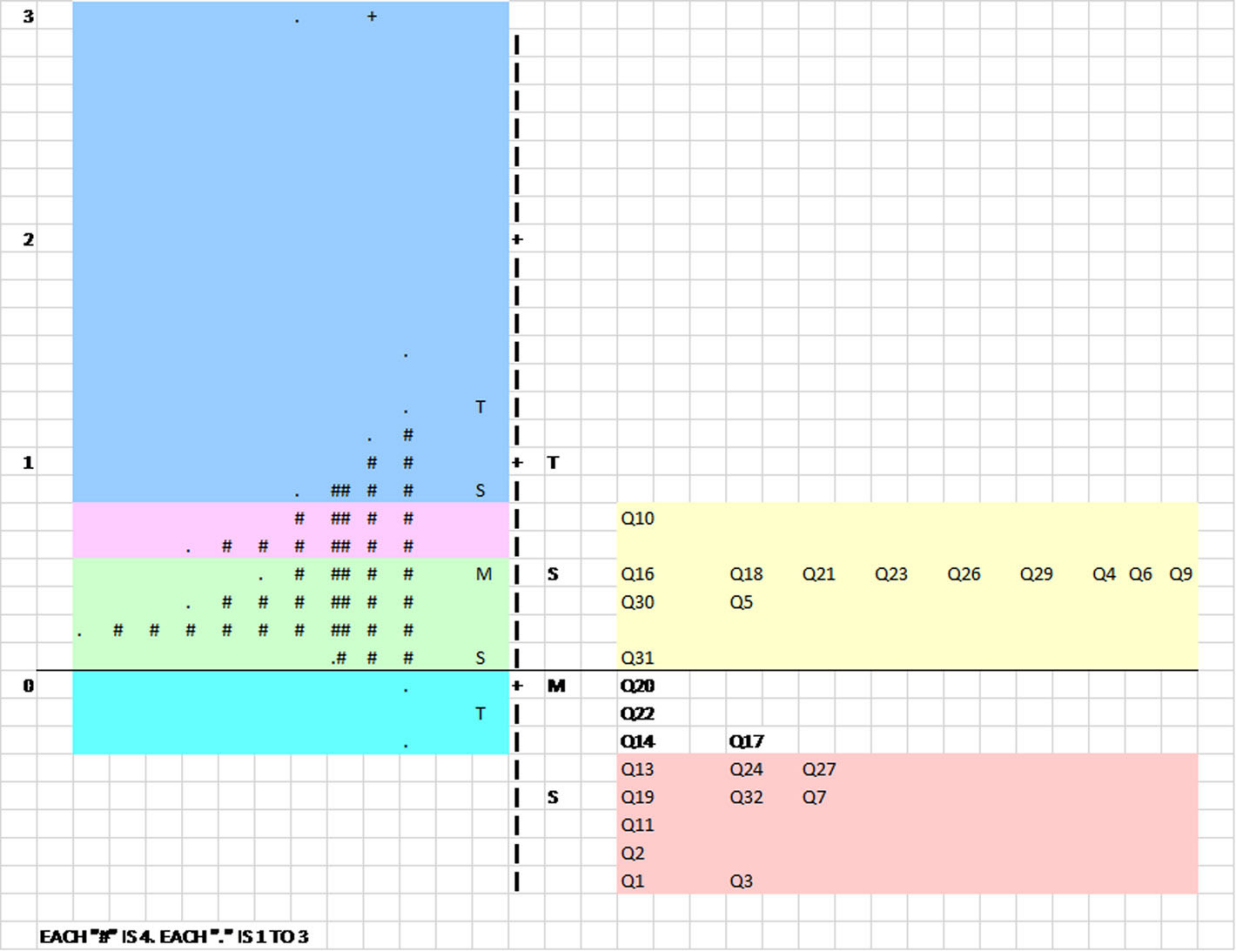
Person-item differential map of labour satisfaction scale

Figure 1 shows the number of respondent ability and item difficulty on the logit scale. All the items are scattered and point towards the ability of respondents’ diversity. The Item mean is set by default at μ_mean_ 0.00 *logit* to ensure that each group of persons has a 50:50 chance of success in responding to the item that matches their ability, while the Person mean, β_mean_ is at 0.54 *logit*. The Item difficulty measures from + 0.74 to − 0.91 logit with the spread of 1.65 *logit*. Meanwhile, the Person’ ability estimates from + 3.03 to − 0.30 *logit* with the spread of 3.33 *logit*.

Respondents that have more difficulty in endorsing satisfaction were located at the top of the map, while the respondents that were easy to endorse satisfaction were located at the bottom of the map. The most difficult item to endorse satisfaction is item 9 (1.55 *logit*) *‘After my baby was born, I was not given him/her quite as soon as I wanted’* at the upper scale. While at the lower scale, 10 items were easy items and were endorsed by all the respondents as satisfactory. In general, 47 respondents were less satisfied with birth labour (above 0.74 *logit*).

## Discussion

Fit is a quality control principle used to indicate how accurately or predictably data fit the model. This process is crucial to see the fit of the measures of each item to allow for further analysis.

The WOMBLSQ indicates good internal consistency of the person in the scale in measuring a single latent trait or construct. The item reliability indices indicate that the items are very good as the values are close to 1.0. The high item reliability also indicates that the 98% likelihood of replicability of the items would occur if these instrument to be given to another sample of respondents of the spectrum [14]. The Person reliability too slightly matches the expected model.

The Item separation index is seven. and this reveals that the 27 items have a good spread in measuring the level of birth labour satisfaction. Furthermore, it indicates that the tool is measuring what it is supposed to measure and, hence, its validity. Furthermore, the items are capable of separating the persons into two groups. Higher values of item separation index indicate a better separation of varying difficulty. The separation is dependent on item reliability. In the cases when misfit persons are identified, it is managed by removing them from the analysis or more persons added. These indices show the ability to measure the person differences of the tool [14, 23].

Unidimensionality is low but fulfilled the minimum threshold of 40%, which is an indication of a strong measurement dimension [24]. This reason is due to the noise in the item which is 10.0%. The unexplained variance in the *1st* contrast between 10 and 15% is a fair indicator of unidimensionality [20]. Therefore, one can conclude that the current 27 items in measuring the level of birth level satisfaction can be treated as unidimensional.

High positive residual correlations may indicate local item dependency between pairs of items or persons. Local dependence tests for the largest standardised residual correlation yields a very good outcome, where none of the items breach the 0.70 limit. This shows that the questionnaire is unidimensional with good internal consistency and is a stable tool measuring what it meant to be measured.

The Item fit statistics showed that outfit MnSq and outfit z-std is at the expectation of 1.00 and 0.00. This gives an indication of the goodness of fit of the instrument, and that it is measuring what is to be measured. In short, the data fits the measurement model. The Item misfit order for item 6 (*‘My birth partner/husband couldn’t have supported me any better’)and* and item 5 *(My birth partner/husband helped me to understand what was going on when I was in labour)* were misfit. The MnSq indicates that the items do not contribute in the construction of scale but not degrading. The PtMea Corr for item 6 was negative indicating the item need to be re-examined for removal or rephrasing as it has elicited careless responses while for item 5 was positive indicating that the item correlates poorly with the construct. The item was maintained but further refining is probably required. The Person fit statistics revealed that the 27 items were targeting the right type of respondents, had little distortion in measuring the latent traits and the produced data was at a reasonable prediction level of the responses to the items.

Wright distribution map represents item difficulty locations and distribution of person along the logit scale. It assists in locating the area where most items are located particularly to see whether this is parallel with the spread of the respondents. Optimal targeting occurs when a set of items in a domain are able to cover the full range of scale score in the population. The mean of the item difficulty should be close to the mean of scale score of the respondents and greater difference in the means leads to poorer targeting [25]. The mean for both respondent ability and item difficulty measurements are approximately around the same location, thus indicating that the items for this sample are well targeted. The item distribution has a much lower spread compared to person spread. The most difficult item to endorse satisfaction is Item 9 *‘After my baby was born, I was not given him/her quite as soon as I wanted’* and 10 items were endorsed by all the respondents as satisfactory.

A potential drawback of this study is that the women were interviewed in the Malay language, which may the limit its generalizability to other populations. Second, the study needs to be repeated in a random sample in other states to be truly representative of a national population. However, this study marks the first attempt at a Rasch analysis of multiple-choice items that measure women’s ability to assess their satisfaction related to childbirth care. All items have an acceptable model fit and can be used in the settings where they were tested.

## Conclusion

The WOMBLSQ tested among mothers following childbirth has been shown to have both a high person reliability index and a high item reliability index. Ten items can easily endorse satisfaction from the respondents. There is no difference in the person reliability after removing eight misfit responses. The item spread is narrow compared to the person spread. For future improvement, a homogenous spread between difficult and easy items should be considered in the construction of items.

## Supplementary information

**Additional file 1.** Women’s Views of Birth Labour Satisfaction Questionnaire in English and Malay languages.

## Data Availability

The authors are happy to share anonymized data related to this paper upon receiving a specific request, along with the purpose of that request. Interested parties may contact hayatikk@usm.my

## Abbreviations

MnSq: outfit mean square
LID: local item dependency
PCA: Principal Component Analysis
PtMea Corr: point-measure correlation
SE: standard error
WOMBLSQ: Women’s Views of Birth Labour Satisfaction Questionnaire
z-std: outfit z-score standardized.
