# Supplementary material for: Psychometric properties of the Malay version of the Women’s Views of Birth Labour Satisfaction Questionnaire using the Rasch measurement model: a cross sectional study
Source: BMC Pregnancy Childbirth. 2020 May 14;20:295. doi: 10.1186/s12884-020-02975-z (PMC7227260; doi:10.1186/s12884-020-02975-z)
Supplement: Supplementary file 1 — Additional file 1. Women’s Views of Birth Labour Satisfaction Questionnaire in English and Malay languages. [file 12884_2020_2975_MOESM1_ESM.pdf]

WOMEN'S VIEWS OF BIRTH LABOUR SATISFACTION QUESTIONNAIRE
SOAL SELIDIK PANDANGAN WANITA TERHADAP KEPUASAN SEMASA BERSALIN

Instruction:
Arahan:
For each of the following statement, please indicate how it relates to you based on the following scale:
Untuk setiap penyataan, sila tandakan bagaimana ia menggambarkan keadaan anda:

Item	*Description*	*Strongly agree*	*Agree*	*Slightly agree*	*Undecided*	*Slightly disagree*	*Disagree*	*Strongly disagree*
Item	Penerangan	Sangat setuju	Setuju	Kurang bersetuju	Tidak pasti	Kurang tidak bersetuju	Tidak bersetuju	Sangat tidak bersetuju
Item 1 (EXP_Q1)	*My labour went totally normally* Proses bersalin saya berjalan secara normal							
Item 5 (HS_Q2)	*My birth partner/husband helped me to understand what was going on when I was in labour* Suami saya membantu saya memahami apa yang sedang berlaku semasa proses bersalin							
Item 8 (HB_Q3)	*I got to see my baby at exactly the right time after she/he was born* Saya dapat melihat bayi saya sejurus selepas dilahirkan							
Item 11 (ENV_Q4)	*My birth room was a little impersonal and clinical* Bilik bersalin saya agak terbuka							
Item 13 (CON_Q5)	*At the start of my labour I knew my carers very well* Dari awal proses bersalin, saya sudah mengenali staf kesihatan dengan rapat							
Item 15 (PAD_Q6)	*I should have been offered something more to relieve the pains I had after my baby was born* Saya sepatutnya diberikan lebih ubat untuk mengurangkan sakit selepas bayi dilahirkan							
Item 21 (PS_Q7)	*All my labour carers were very supportive* Semua staf memberi sokongan yang penuh kepada saya semasa mengendalikan proses bersalin							
Item 18 (LP_Q9)	*I should have been offered something more to relieve my labour pains* Saya sepatutnya ditawarkan sesuatu untuk mengurangkan sakit bersalin							

Item 9 (HB_Q10)	*After my baby was born, I was not given him/her quite as soon as I wanted*<br>Selepas bayi saya dilahirkan, dia tidak diberikan kepada saya secepat yang dikehendaki							
Item 2 (EXP_Q11)	*The labour went nearly exactly as I had hoped that it would*<br>Proses bersalin saya berjalan lancar seperti mana yang diharapkan							
Item 22 (PS_Q13)	*Carers always listened very, very carefully to everything that I had to say*<br>Staf kesihatan sentiasa mendengar dengan teliti apa yang ingin saya sampaikan							
Item 12 (ENV_Q14)	*The area where I gave birth was very pleasant and relaxing*<br>Tempat saya melahirkan bayi sangat selesa dan tenteram							
Item 16 (PAD_Q16)	*I was in a fair bit of pain immediately after the birth*<br>Saya berada dalam keadaan yang agak sakit sejurus selepas melahirkan bayi							
Item 3 (EXP_Q17)	*The delivery went almost completely as I had hoped that it would*<br>Proses kelahiran bayi berjalan lancar seperti mana yang diharapkan							
Item 10 (HB_Q18)	*I needed to hold my baby a little earlier than I did*<br>Saya perlu memegang bayi saya lebih awal							
Item 23 (PS_Q19)	*During labour there was always a carer to explain things so that I could understand*<br>Semasa proses bersalin, ada staf yang sentiasa memberi penerangan berkenaan apa yang akan berlaku seterusnya untuk memastikan saya faham							
Item 19 (LP_Q20)	*I got excellent pain relief in labour*<br>Saya mendapat ubat penahan kesakitan yang baik semasa bersalin							
Item 26 (CTRL_Q21)	*Everyone seemed to tell me what to do in labour*<br>Setiap orang seolah-olah memberitahu saya apa yang sepatutnya dilakukan semasa proses bersalin							
Item 4 (EXP_Q22)	*My labour was just about the right length*<br>Proses bersalin berlaku							

	dalam tempoh yang sepatutnya							
Item 6 (HS_Q23)	*My birth partner/husband couldn’t have supported me any better* Suami saya tidak dapat memberikan sokongan yang lebih baik daripada apa yang telah diberikan							
Item 14 (CON_Q24)	*I knew the carer(s) present at the birth of my baby* Saya tahu staf kesihatan akan berada di samping saya semasa melahirkan bayi							
Item 20 (LP_Q26)	*More pain relief would have made my labour easier* Jika diberikan ubat penahan kesakitan yang lebih, proses bersalin saya akan menjadi lebih mudah							
Item 24 (PS_Q27)	*All my carers treated me in the most friendly and courteous manner possible* Semua staf kesihatan melayan saya dengan cara yang sangat ramah dan bersopan							
Item 7 (HS_Q29)	*I could have had a bit more help from my birth partner/husband* Saya sepatutnya mendapat lebih bantuan daripada suami saya							
Item 27 (CTRL_Q30)	*Labour was just a matter of doing what I was told by my carers* Proses meneran adalah proses melakukan apa yang telah diajar oleh staf kesihatan kepada saya							
Item 17 (PAD_Q31)	*I didn’t need a lot of pain relief after the birth* Saya tidak memerlukan banyak ubat tahan sakit selepas melahirkan bayi							
Item 25 (PS_Q32)	*My carers couldn’t have been more helpful* Staf kesihatan sudah membantu saya dengan sepenuhnya							
